# The state of research on cyberattacks against hospitals and available best practice recommendations: a scoping review

**DOI:** 10.1186/s12911-018-0724-5

**Published:** 2019-01-11

**Authors:** Salem T. Argaw, Nefti-Eboni Bempong, Bruce Eshaya-Chauvin, Antoine Flahault

**Affiliations:** Institute of Global Health, Faculty of Medicine, University of Geneva, Campus Biotech, Chemin des Mines 9, 1202 Geneva, Switzerland

**Keywords:** Cyberattacks, Hospital cybersecurity, Medical device security, Cybersecurity recommendations

## Abstract

**Background:**

The health sector has quickly become a target for cyberattacks. Hospitals are especially sensitive to these sorts of attacks as any disruption in operations or even disclosure of patient personal information can have far-reaching consequences. The objective of this study was to map the available literature on cyberattacks on hospitals and to identify the different domains of research, while extracting the recommendations and guidelines put forth in the literature.

**Methods:**

Four databases (PubMed, Web of Science, ProQuest, and Scopus) were searched using standardized and adapted search syntax in order to identify relevant manuscripts published between 1997 and 2017. These were screened by two reviewers and included or excluded based on inclusion and exclusion criteria. Data from articles were then extracted and analyzed.

**Results:**

The search identified 818 records of which 97 were included. Of the 97, 32% were published in 2017 while around 40% of the articles were published prior to the last three years. Six domains of research emerged through the analysis, which are included here: context and trends in cybersecurity (27.8%), connected medical devices and equipment (29.9%), hospital information systems (14.4%), raising awareness and lessons learned (6.2%), information security methodology (15.4%), and specific types of attacks (6.2%).

**Conclusion:**

There is a generally growing interest in the research field, but the available literature remains limited in number. There are important aspects of cybersecurity (e.g. cloud storage and access management) as well as specific medical fields that rely on various medical devices that have been neglected. Recommendations are available, but comprehensive guidelines and standardized best practice measures are still necessary.

**Electronic supplementary material:**

The online version of this article (10.1186/s12911-018-0724-5) contains supplementary material, which is available to authorized users.

## Background

Violence against hospitals—manifested in physical attacks against patients, workers, and facilities [1] as well as in cyberattacks on hospitals—has been on the rise worldwide. Cyber violence has especially become rampant in recent years, affecting numerous hospitals in high-income countries such as the United States (US) [2, 3] as well as Norway [4] and even becoming a concern for lower-middle income countries such as Kenya [5]. Cyberattacks include a variety of threats from brute force and Denial-of-Service attacks to the use of phishing and malware or social engineering methods to compromise security [6].

Whilst a ransomware is only one type of malware threatening health facilities, a report by the US Department of Justice revealed that an average of 4000 ransomware attacks occurred daily across different sectors in 2016—a 300% increase since 2015 [7]. Another report revealed that the health field was among the top three sectors most affected by ransomware worldwide [8]. Besides ransomware, there has also been a four-fold increase in the number of malicious computer software attacks in the last two years [9] and the health sector has become one of the most targeted sectors globally [10].

This is a growing concern as hospitals worldwide are becoming increasingly dependent on their hospital information systems for administrative, financial, and medical operations—with the use of connected medical devices, cloud storage services, and network systems simultaneous rising. There is a widespread understanding of the need to balance utility and efficacy with privacy and security in innovation; however, technology is bolstering more quickly than the creation, application, and update of security measures [11]. The reality is that healthcare is lagging behind other sectors in securing data as well as in developing comprehensive employee training programs—even if findings show the latter as the most stressed strategy against breaches in the literature [11–14].

Additionally, the healthcare sector is especially vulnerable to attacks because the nature of the work makes it extremely sensitive to any disruption in its services. A delay in hospital operations—much less a halt—can have devastating consequences on patient safety. A ransomware attack on Hollywood Presbyterian Medical Center in Los Angeles caused cancellations of procedures and redirection of in-coming ambulances over the span of 10 days [15, 16]. A different attack, on the British National Health Service (NHS), had similar effects on hospitals as well as additional debilitating effects on radiology and blood-product refrigeration of hospitals [16].

These sort of attacks heighten risks to patient safety as providers lose access to virtual records of comorbidities, allergies, and existing prescriptions [6, 7]. In addition to these effects on health delivery, breach and disclosure of sensitive health information can have detrimental effects on an individual’s social and professional life [17] as well as expose the patient to the risks of blackmailing [18]. Moreover, cybercriminals can commit a range of crimes from identity theft to medical fraud with patients’ personally identifiable information [11]. At the other end, the financial consequences to hospitals are substantial with direct costs from patient compensation and regulation fines as well as the long-term financial consequences that follow damage to their facilities’ reputation [19]. On a larger scale, the consequences of interrupted care delivery can affect the larger hospital network (i.e. spreading into ambulance, pharmacy, and health insurance company operations).

When considering the motives behind such attacks, financial gain is a reoccurring topic. The information accessed through health data breaches is of particular interest as it is highly valued on the dark web [20]; medical records are even worth more than social security numbers [21]. Additionally, since these records include dates of birth, residential addresses, and health information, the stolen data is durable and widely applicable to criminal activities [10]. Other motives include nation state (state sponsored), terrorist, retribution, and hacktivist interests [6] and sources of attacks can be external with local or remote actors and internal through deliberate or inadvertent acts [4].

The objective of this scoping review was to map the available peer-reviewed literature focused on cyberattacks against hospitals from the past two decades as well as to identify the different domains of research investigated in the literature, while considering the recommendations and guidelines put forth for the multidisciplinary community of concern (e.g. manufacturers, hospital managers, clinicians, patients, policy-makers, and government agencies). This review was the first step towards developing the focus and direction of the *seventh edition Geneva Health Forum M8 Alliance Expert Meeting* working group’s efforts on identifying the challenges and mitigating the risks of cyberattacks on hospitals. The research questions of this scoping review were as follows:

1. What are the domains of research previously emphasized in the literature on cybersecurity of hospitals?
2. What recommendations are put forth by the available literature?

## Methods

### Overview

This review was conducted using the methodological framework for scoping reviews proposed by Arksey and O’Malley [22] in conjunction with the advancements recommended by Levac et al. [23]. While this scoping review is not registered, it adheres to the Preferred Reporting Items for Systematic Reviews and Meta-Analyses (PRISMA) Guidelines [24] where applicable.

Within this review, discussion of cybersecurity did not include non-malicious cybersecurity breaches. The focus was to concentrate specifically on cyber threats and cyberattacks targeted against hospitals. Additionally, the cybersecurity of hospitals included the cybersecurity of connected medical devices, hospital information systems (HIS), and health records. However, publications related to mHealth cybersecurity were considered out of the scope of this review, as mobile devices do not directly constitute the hospital infrastructure.

### Search strategy

In order to broadly capture existing work, relevant literature was gathered using the following four search engines (pertinent databases have been listed in conjunction): PubMed (MEDLINE), Web of Science, ProQuest (CINAHL), and Scopus (EMBASE, Compendex). These were selected after brief analysis of previous work and preliminary search results. Searches were initially conducted in October 2017 and later updated in March 2018.

The methodology began with the identification of pertinent key terms and pre-existing keywords through preliminary searches. Terms related to hospitals, medical devices, HIS, electronic health records (EHR), and electronic medical records (EMR) were gathered along with terms pertaining to cyberattacks and cyber threats. Pertinent terms were selected after two separate internal discussions and then strung together with Boolean operators ([AND], [OR]). After several rounds of trial and error as well as discussion, a search syntax was established and then adapted to each database (see Additional file 1 for an example). Other records were later identified and included through methods such as hand searching and snowballing.

### Selection of studies

After the identification phase of the PRISMA four-phase flow diagram [24], two reviewers separately screened all records for relevance using bibliographic data (i.e. title, type of publications, abstracts). Duplicated records were excluded and full-texts were then retrieved for review. If full-text versions were not initially available, articles were acquired through the interlibrary loan system.

The inclusion and exclusion criteria, which had been discussed and established following the preliminary search, were then applied to assess eligibility. Records in English published between January 01, 1997 and December 31, 2017 in peer-reviewed journals were selected for inclusion. Study location or country of publication did not affect inclusion—apart from country biases that may already exist in the databases. Primary literature, literature reviews, and editorial material as well as conference proceedings published in peer-reviewed journals were all included. Studies reporting qualitative and quantitative findings were equally selected.

**Fig. 1:**
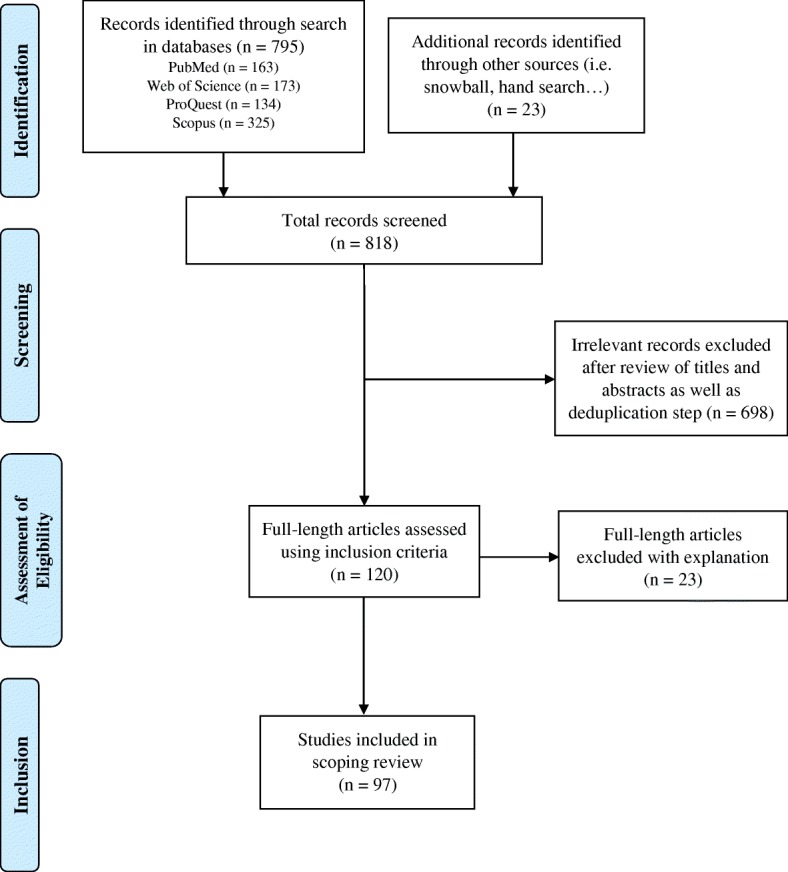
Study Selection Process in accordance with PRISMA guidelines

Articles were excluded after full-text evaluation because of the following reasons: they did not discuss cyberattacks in relation to the hospital setting or they were focused on other forms of security outside of cyber. (The study selection process is illustrated in Fig. 1).

### Data collection, extraction, and analysis

The full-texts of the selected articles were read and relevant data was then extracted into a standardized data extraction chart. Extracted data items included the source of the article, study design, title, author(s), year of publication, journal, author(s)‘s affiliation(s), funding source(s), area of focus, important findings, and pertinent recommendations. The aim of each study as well as a summary of the article was also integrated into the data extraction chart. Citations were organized using the Mendeley Reference Management Software.

The selected articles were categorized based on their study design after operational definitions were established (see Additional file 2). Additionally, the theme of the 97 articles were identified through analysis of the full-texts. These themes were then classified in order to identify common research domains. These domains were based upon exiting research topics in the field but were developed to capture the entirety of the scope of the field’s literature into concise categories.

## Results

### Overview

The literature identification phase resulted in 818 records: 795 (163 from PubMed, 173 from Web of Science, 134 from ProQuest, and 325 from Scopus) were identified through the selected databases before the deduplication step and 23 records were later identified through methods such as hand searching and snowballing. These records were screened, unduplicated, and assessed for eligibility. Subsequently, 97 articles were selected for inclusion into the review (see Fig. 1). Publications included in this scoping review have been marked with an asterisk (*) in the references section.

Investigation into funding sources revealed that 21 of the publications (21.6%) received at least part of their funding from governmental agencies such as the National Science Foundation or National Institutes of Health, while three publications used funding from companies, personal resources, or university consortiums. 74 of the 97 (76.3%) claimed no funding sources or did not report any. (See Additional file 3.) This was followed up with an analysis of the researchers behind the publications. It was found that universities and other teaching institutions were at least partly involved in the publication of 71 articles, while organizations and companies were at least partly behind 14 articles. The remaining articles came at least partly from researchers at insurance companies, government agencies, law firms, and research institutions among other sources (see Additional file 4).

**Fig. 2:**
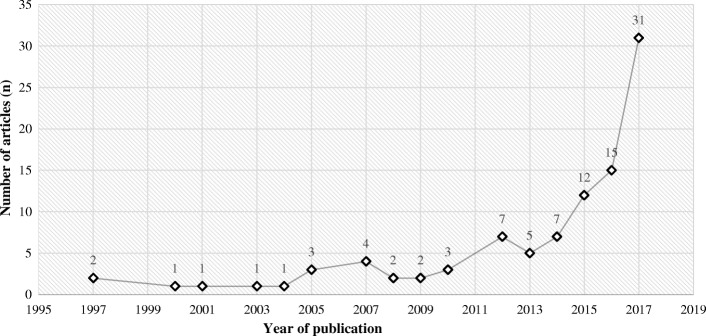
Yearly distribution of publications

Of the 97 articles, 31 were published in 2017, 15 in 2016, and 12 in 2015. From 1997 to 2010, there was less than or equal to four articles published per year. After 2005, there was a slow increase in publication rate, which then skyrocketed after 2012 (see Fig. 2). The majority (59.8%) of the literature was published in the last three years with at least two articles dating back to 1997. Thirty-two of the 97 articles offered practical recommendations and guidelines, often drawing from agencies and institutes such as the U.S. Food and Drug Administration (FDA), the International Electrotechnical Commission (IEC), and the Institute of Electrical and Electronics Engineers (IEEE).

### Medical specialties

**Fig. 3:**
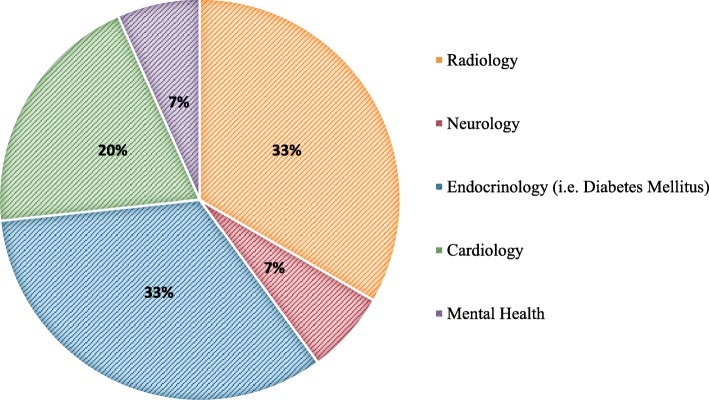
Publications concentrated on the cybersecurity of specific medical specialties

Fifteen of the selected articles (15.5%) were focused on cybersecurity as it relates specifically to certain medical specialties. Devices used in endocrinology—especially those used in the treatment of diabetes mellitus—represented one-third of these articles as did radiology. The cybersecurity of other devices and systems in the fields of neurology, cardiology, and mental health are also explored, but to a lesser extent (see Fig. 3).

### Study designs and identified research domains

The literature is composed of various study designs. Ten of the articles (10.3%) are descriptive studies, 31 (31.9%) are summative reports, 28 (28.9%) are editorials, 20 (20.6%) are technical papers, 5 (5.2%) are literary reviews, and 3 (3.1%) are experimental studies. (See Additional file 2 for details and operational definitions).

Further analysis of the selected literature led to the development of six domains of research: context and trends in cybersecurity, connected medical devices and equipment, hospital information systems, raising awareness and lessons learned, information security methodology, and specific types of attacks (i.e. ransomware, phishing, and social engineering attacks). These are further examined below. The link between the different domains of research and the study designs are illustrated in Table 1.

**Table 1:** Identified domains of research and types of studies

Research domain	Brief description of research domain	Number of articles	Study design with reference
Context and trends in cybersecurity	Explores context of the field, formulates definitions of pertinent terms, offers generalized recommendations, and describes trends in cybersecurity.	27	- Literature review [11, 21] - Descriptive study [27, 29, 68, 78] - Summative report [28, 30, 65, 66, 70, 77, 79, 80] - Editorial [10, 16, 25, 26, 67, 81–87] - Technical paper [88]
Connected medical devices and equipment	Discusses the development, research, and security of connected medical devices and equipment (includes implantable and wearable devices found in neurology, cardiology, endocrinology, mental health, and radiology)	29	- Summative report [6, 31, 33, 37, 38, 50, 75, 76, 89–94] - Editorial [32, 39, 74, 95–99] - Experimental study [40, 41] - Technical paper [34–36, 100, 101]
Hospital information systems (HIS)	Offers methods for evaluating HIS, discusses security concerns of electronic health records, and proposes specific recommendations. Also includes discussions on data security and cloud-based storage.	14	- Descriptive study [44, 102] - Literature review [18, 45, 103] - Summative report [9, 42, 43, 47, 104] - Technical paper [48, 49] - Editorial [46] - Experimental study [105]
Raising awareness and lessons learned	Discusses previous attacks and lessons learned, as well as training programs for various players. Also proposes and evaluates methods for the dissemination of information.	6	- Descriptive study [53, 54] - Editorial [14, 51, 52, 55]
Information security methodology	Discusses network security, multifactor authentication, encryption, password protection, updates and others.	15	- Technical paper [56–58, 60–62, 71–73, 106–108] - Summative report [59, 109, 110]
Specific types of attacks (i.e. ransomware, phishing, and social engineering attacks)	Offers definitions, background information, and recommendations specific to these attack types in the context of hospitals.	6	- Descriptive study [111, 112] - Editorial [15, 63, 64] - Summative report [69]

### Domains of research

#### Context and trends in cybersecurity

A subset (27.8%) of the literature offers generalized discussion and description of cyber threats and attacks—the terms associated with the field, infrastructure of cyber defense (in terms of agencies and regulations)—and offers strategies for security. These articles track the evolution of cybercrime in the health field [21, 25] while exploring some motives for attacks, such as monetary drivers [26], and discussing probable consequences and challenges associated with the cybersecurity of hospitals [27]. Among the challenges raised is the limited budget of hospitals and the eminent priority to provide care to patients [10, 28]. One article in particular discusses patient’s continued trust in the healthcare system and the increasing frequency of cyberattacks [26]. There is also discussion on the effect of compartmentalization of nation’s healthcare systems into public and private entities, and how this affects security and differs from other public health surveillance systems [10, 26, 29, 30]. Several general recommendations are also made for hospitals [10, 16, 28, 30]. The articles in this domain include literature reviews, descriptive studies, summative reports, editorials, and a technical paper.

#### Connected medical devices and equipment

The security of connected medical devices and equipment was a major focus in the literature (29.9%). Study designs vary from experimental studies, technical papers, summative reports, and editorials. The articles discuss the FDA pre-market and post-market guidelines for medical devices, the British Standards Institution recommendations, and relevant IEC documents among several other sources of recommendations and guidelines [31, 32]. In addition, some of the articles propose frameworks for cybersecurity and blueprints for value-based presentation of security measures in the lifecycle and development of medical devices [33–36]. Others discuss the tradeoffs of safety and security with availability and utility when weighting the advantages to patients’ quality of life with associated risks of connected medical devices [37, 38]. There is also discussions on the shared—as well as individual—responsibilities of manufacturers and user facilities for the security of these device [31, 34, 36, 39, 40]. In addition, some articles discuss the various sources of vulnerabilities in devices and equipment and analyze different cybersecurity research methods [6, 41].

#### Hospital information systems

Publications within this research domain encompasses 14.4% of the literature body. It includes articles from all of the study design categories identified in the review. The security concerns of HIS are discussed as well as the challenges of using widespread health information technology [42, 43]. One article presents a model for evaluating and comparing HIS at different hospitals and applies it to local healthcare facilities in Iran [44]. Other articles propose security techniques for EHR systems such as various types of firewalls, cryptography and cloud computing methodologies among others [45, 46]. Data security, storage, and specifically cloud-based storage are also prevalent topics of discussion in this domain [45–49]. The literature presents data storage requirement and recommendations including but not limited to confidentiality and access control, integrity of data, availability and performance, and support for long retention and secure migration [46].

#### Raising awareness and lessons learned

With research revealing that humans are among the weakest link in cybersecurity [35, 50], the importance of raising awareness among end users is stressed throughout domains. Six (6.2%) of the selected articles—descriptive studies and editorials—focus specifically on this topic. A significant portion of the discussion in this domain is on previous attacks [51, 52] and lessons learned—emphasizing the importance of information sharing methodology [53]. Practical recommendations were also proposed for end users such as always changing the password on new devices, using strong passwords, refraining from leaving devices unattended, and avoiding connecting to public WiFi services [14]. More generally, hospitals are advised to enact mock exercises for providers and other hospital staff annually—integrating lessons learned from recent attacks [14]. In one article, researchers used a vocabulary and scenario-based test to evaluate hospital staff’s current understanding of cybersecurity in order to offer relevant and appropriate training [54]. While these sorts of engaging practices are recommended in order to keep staff vigilant, it is crucial for hospitals to have the leadership, governance, and information technology (IT) staff for cybersecurity [55].

#### Information security methodology

The third most prevalent research domain—making up 15.4% of the literature body—is information security methodology. These publications are technical papers and summative reports. Definitions, methods, and general information are discussed on a wide array of specific topics such as network security, multifactor authentication, security of medical imaging, password protection, and patching systems [56–59]. Recommendations and techniques are also proposed in these articles for reducing data leakage [60], strengthening end users’ passwords [61], as well as utilizing intrusion prevention systems [62].

#### Specific types of attacks (i.e. ransomware, phishing, and social engineering attacks)

A small portion (6.2%) of the selected articles focus specifically on phishing, social engineering, and ransomware attacks. These articles are summative reports, descriptive studies, and several editorials that focus on describing and defining the attack type, recounting previous episodes of attacks, and proposing recommendations for mitigating risks. Cybersecurity events such as the February 2016 Hollywood Presbyterian Medical Center ransomware attack in Los Angeles, California and the March 2016 Medstar Health ransomware attack in Maryland, Baltimore as well as several phishing attacks are explored [63, 64].

### Recommendations

Among the recommendations found in the 97 selected articles, researchers state that organizations should allocate more resources and funding to IT security [18, 57] and should define the cybersecurity duties of employees [11]. In addition, key guidelines are put forth that recommend risk assessment methods, intrusion prevention services and penetration testing, loss of data as well as log monitoring systems, firewall implementation, network auditing, and privilege restrictions as well as methods for regularly checking critical server files [30, 65].

On the topic of connected medical devices, recommendations were put forth for companies to implement reasonable measures such as access control on devices and security testing beyond the developmental phase [66]. They are also asked to enforce antivirus scans and the use of firewalls [40], and to have reporting mechanisms that users can apply to communicate cybersecurity issues [67]. Regulators are asked to propose best practices, but balance encouraging security with burdensome regulations [68]. Other researchers propose an independent nonprofit organization composed of medical, industry, and academic experts be charged with developing standards for device cybersecurity [66]. Furthermore, providers are advised to collaborate with security experts and to hold high standards of security research methods [41]. Additional recommendations emphasize information sharing systems as well as hospital risk management and contingency planning that takes medical devices into account [31].

Hospitals are additionally advised to develop training programs that are at least annually re-evaluated and amended based on recent events. Training is recommended in privacy policies, data leakage prevention, and workplace social media use, but is especially stressed in digital hygiene—good practices of digital security such as choosing strict privacy settings and strong password protection. End users should not use the same password for multiple accounts, trust suspicious emails, or leave computers unattended. Healthcare organizations are also advised to set and enforce proper policy for password protection and sharing of information. These training programs are to be developed with consideration of the perspectives and level of digital experience of a multidisciplinary team (i.e. providers, IT specialists, hospital managers) in order to truly be effective [14]. It is also recommended that hospitals should run IT security drills and mock system recovery exercises in order to keep all members vigilant [65, 69].

Specific recommendations are made for phishing attempts and social engineering attacks such as the need for specialized training programs for these attack types and others, filtering of both emails and websites, enforcement of frequent password changes—at the cost of convenience—and reasonable limitations on access to data [64]. Similar recommendations were put forth for ransomware attacks along with regular and (ideally, real-time offline) backups, encryption of sensitive data, and technical safeguards such as up-to-date antivirus software, automated patches, pop-up blockers, and prevention of USB usage [63, 69]. Along these lines, de-identification and data encryption, minimization of requested data, and deletion of unnecessary data are emphasized all throughout. In addition, the literature recommends timely update of third-party software and strict limits on downloads from untrusted sources. The possibility of national level health data warehouses was also discussed, but with a focus on the security and privacy measures that would be required beforehand [18].

## Discussion

There is an increase in the pace of publication following 2012 with an exponential rise after 2016. This escalation in research pace may be related to the 2016 Hollywood Presbyterian ransomware attack, which was the first highly publicized cyberattack incident against a hospital. There were several other incidents following this, but the importance of cybersecurity in hospitals took the headlines once again in May 2017 when the WannaCry ransomware attack affected the NHS hospitals. There is reason to believe that the rate of publications will continue to grow as hospitals turn their attention to fortifying their cybersecurity systems [9, 26].

The review also revealed some breadth in the research field with publications focusing on various research domains (six identified) and medical specialties, but an overall lack of quantity of available literature. For instance, while topics such as health data encryption are explored, methodological and more real-world operational studies on the topic would have been expected. Other areas of research that were neglected in relation to the cybersecurity of connected medical devices, for example, are security of cloud storage, the use of USB ports, and the topic of identity and access management as well as ethics. Similarly, while medical specialties such as neurology [50], radiology [40, 70–73], cardiology [41, 48, 74], endocrinology [25, 32, 33, 75, 76], and mental health [77] are represented, they are in the minority of the selected articles.

Analysis of study designs also demonstrates a large proportion of editorials (nearly 29%) and summative reports (nearly 32%). These documents focused on raising awareness and the general knowledge base of readers. This reveals that the target audience of these publications was a broad group of actors non-specialized in information security (i.e. clinicians, hospital administration staff, management teams, and policymakers). This finding illustrates that cybersecurity of hospitals is the concern of a larger and multidisciplinary group and that security measures cannot be successful without the active participation of the various professionals in a hospital. The finding also indicates that there may be further need for methodological studies specifically in information security.

Investigation into funding sources was made to explore conflicts of interests or the presence and possible influence of companies and industry representatives in the research field. However, companies directly funded only 1% of the publications and only around 14% of the research was at least partly conducted by companies or organizations. Nevertheless, there may be a stronger presence of cybersecurity companies, for example, in the research field than is depicted in academic, peer-reviewed journals, which was the scope of this paper.

Of the six research domains that emerged in this scoping review, the cybersecurity of connected medical devices and equipment presented as the most prevalent research area in the literature (29.9% of the selected articles). This may be as the security of connected devices is particularly challenging. These devices are discrete systems that necessitate integration into the hospital IT infrastructure and become ubiquitous in the network, but that often lack inherent protection in the form of firewall or antivirus due to power supply limitations [6, 11]. Connected devices and equipment are a large part of hospital operations and thus, their security is a vital topic.

### Limitations

This review provides an up-to-date analysis on current research domains and available recommendations on the cybersecurity of hospitals. While there are two systematic reviews [11, 21] previously published in the field of cybersecurity in healthcare, they were focused on identifying trends in the number of cyberattacks on healthcare and themes in cybercrime, respectively. There has not been a review that investigates the body of literature on cyberattacks against hospitals or that assembles the recommendations put forth. This scoping review yielded an overview of the relatively new and quickly growing literature body and was able to cope with the pace of publications. A systematic follow-up could expand on these research domains and refine subject headings further.

There are a few limitations to this study. Selection of publications was limited to articles available in the English language, which could have excluded several important publications from countries with advanced cybersecurity methodology. Along the same lines, articles in peer-reviewed journals not listed in the four databases selected could have also been unintentionally excluded. This is also a field in which industry representatives outside of academia are highly engaged and limiting the review’s scope to peer-reviewed academic journals could have excluded additional relevant publications.

## Conclusion

The objective of this study was to identify and map the scientific literature on cyberattacks on hospitals and to describe the different areas of research in this literature. Six domains of research (context and trends in cybersecurity, connected medical devices and equipment, hospital information systems, raising awareness and lessons learned, information security methodology, and specific types of attacks) were developed to map the literature. These domains of research can be refined and developed by the field in the future. While 97 articles were identified from the past two decades and studied, it was apparent that there was an overall lack of quantity in publications. However, the review indicated a generally growing interest in the production of studies and recommendations on the topic. As the frequency of cyber threats continues to grow, the value of comprehensive guidelines and standardized best practice measures will become unequivocal.

## Additional files

Additional file 1: **Table S2.** Example of search strategy syntax. Search syntax used for PubMed provided to give readers example of how Boolean operators were used to string together search terms. (DOCX 13 kb)

Additional file 2: **Table S3.** Types of studies and operational definitions. Lists the operational definitions for study types used by authors in conducting the study (DOCX 12 kb)

Additional file 3: **Table S4.** Funding sources. Details which manuscripts had which funding sources (DOCX 12 kb)

Additional file 4: **Table S5.** Where is the research coming from? Details which manuscripts came from what type of institute (DOCX 12 kb)

## Abbreviations

EHR: Electronic Health Records
EMR: Electronic Medical Records
FDA: Food and Drug Administration
HIS: Hospital Information System
IEC: International Electrotechnical Commission
IEEE: Institute of Electrical and Electronics Engineers
IT: Information Technology
NHS: National Health Services
PRISMA: Preferred Reporting Items for Systematic Reviews and Meta-Analysis
US: United States
