# Supplementary material for: The state of research on cyberattacks against hospitals and available best practice recommendations: a scoping review
Source: BMC Med Inform Decis Mak. 2019 Jan 11;19:10. doi: 10.1186/s12911-018-0724-5 (PMC6330387; doi:10.1186/s12911-018-0724-5)
Supplement: Supplementary file 1 — Table S2. Example of search strategy syntax. Search syntax used for PubMed provided to give readers example of how Boolean operators were used to string together search terms. (DOCX 13 kb) [file 12911_2018_724_MOESM1_ESM.docx]

Additional file 1

*Table S2: Example of search strategy syntax*

| **Databases** | **Search Syntax** |
| --- | --- |
| PubMed | ("healthcare"[Title/Abstract] OR "hospital"[Title/Abstract] OR "health facility"[Title/Abstract] OR "hospital administration"[Title/Abstract] OR "hospital organization"[Title/Abstract] OR "health centers"[Title/Abstract] OR "health facility administration"[Title/Abstract] OR "hospital units"[Title/Abstract] OR "clinic"[Title/Abstract] OR "diagnostic devices"[Title/Abstract] OR "diagnostic equipment"[Title/Abstract] OR "medical devices"[Title/Abstract] OR "networked medical devices"[Title/Abstract] OR "medical imaging software"[Title/Abstract] OR "medical equipment"[Title/Abstract] OR "medical monitors"[Title/Abstract] OR "medical monitoring systems"[Title/Abstract] OR "electronic health records"[Title/Abstract] OR "ehr"[Title/Abstract] OR "electronic medical records"[Title/Abstract] OR "emr"[Title/Abstract] OR "health information systems"[Title/Abstract] OR "hospital information systems"[Title/Abstract] OR "medical informatics"[Title/Abstract] OR "medical technology"[Title/Abstract] OR "medical records"[Title/Abstract] OR "patients"[Title/Abstract] OR "patient information"[Title/Abstract]) AND ("cyber attacks"[Title/Abstract] OR "cyber-attacks"[Title/Abstract] OR "cyberattacks"[Title/Abstract] OR "cyber terrorism"[Title/Abstract] OR "cybercrime"[Title/Abstract] OR "cyber crime"[Title/Abstract] OR "cyber threats"[Title/Abstract] OR "compromising of data"[Title/Abstract] OR "computer hackers"[Title/Abstract] OR "hackers"[Title/Abstract] OR "computer viruses"[Title/Abstract] OR "computer worms"[Title/Abstract] OR "ransomware"[Title/Abstract] OR "phishing"[Title/Abstract] OR "data breach"[Title/Abstract] OR "malware"[Title/Abstract] OR "firewall"[Title/Abstract] OR "antivirus"[Title/Abstract] OR "spyware"[Title/Abstract] OR "adware"[Title/Abstract] OR "information warfare"[Title/Abstract] OR "cybersecurity"[Title/Abstract] OR "cyber security"[Title/Abstract]) AND (("1997/01/01"[PDAT] : "2017/12/31"[PDAT]) AND English[lang]) |
