# Supplementary material for: The state of research on cyberattacks against hospitals and available best practice recommendations: a scoping review
Source: BMC Med Inform Decis Mak. 2019 Jan 11;19:10. doi: 10.1186/s12911-018-0724-5 (PMC6330387; doi:10.1186/s12911-018-0724-5)
Supplement: Supplementary file 2 — Table S3. Types of studies and operational definitions. Lists the operational definitions for study types used by authors in conducting the study (DOCX 12 kb) [file 12911_2018_724_MOESM2_ESM.docx]

Additional file 2

*Table 3: Types of studies and operational definitions*

| **Type of study** | **Operational definition** | **Number of studies** |
| --- | --- | --- |
| Descriptive studies | Studies using qualitative analysis, including narrative methods and historical analysis, as well as case studies. | 10 |
| Summative Reports | Literature that aims to provide readers with detailed and descriptive context, information, recommendations, and/or definitions. | 31 |
| Editorials | Articles that offer an opinion and/or a discussion on the topic or short texts aimed at raising general awareness. This can include documents prepared as quick reference guides, flyers for professionals, news reports, opinion papers, question and answer, letters, and viewpoints as long as they were published in peer-reviewed journals. | 28 |
| Technical Papers | Peer-reviewed papers that propose any sort of new technique, but does not involve experiments. | 20 |
| Literature reviews | Literature reviews whether systematic or not. | 5 |
| Experimental studies | Articles in which actually experimental studies were conducted. | 3 |
