# Supplementary material for: The state of research on cyberattacks against hospitals and available best practice recommendations: a scoping review
Source: BMC Med Inform Decis Mak. 2019 Jan 11;19:10. doi: 10.1186/s12911-018-0724-5 (PMC6330387; doi:10.1186/s12911-018-0724-5)
Supplement: Supplementary file 3 — Table S4. Funding sources. Details which manuscripts had which funding sources (DOCX 12 kb) [file 12911_2018_724_MOESM3_ESM.docx]

Additional file 3

*Table 4: Funding sources—at least partly funded through these means.*

| **Source** | **Number of publications** | **Study Reference** |
| --- | --- | --- |
| Government agencies (i.e. NIH or Ministries) | 21 | [19, 28, 34, 36, 48, 50, 56, 57, 61, 63, 71, 72, 74, 75, 78, 88, 90, 92, 100, 101, 111] |
| “None” reported or not reported | 74 | [6, 10, 11, 14–16, 21, 25–27, 29–33, 35, 37–47, 49, 51–55, 58–60, 62, 64–70, 73, 76, 77, 79, 81–87, 89, 91, 93–99, 102–110, 112] |
| University consortium (i.e. SICSA) | 1 | [80] |
| Authors’ resources | 1 | [9] |
| Company | 1 | [90] |
