# Supplementary material for: The state of research on cyberattacks against hospitals and available best practice recommendations: a scoping review
Source: BMC Med Inform Decis Mak. 2019 Jan 11;19:10. doi: 10.1186/s12911-018-0724-5 (PMC6330387; doi:10.1186/s12911-018-0724-5)
Supplement: Supplementary file 4 — Table S5. Where is the research coming from? Details which manuscripts came from what type of institute (DOCX 12 kb) [file 12911_2018_724_MOESM4_ESM.docx]

Additional file 4

*Table 5: Where is the research coming from?*

| **Source of research** | **Number of publications coming at least party from these sources** | **Study Reference** |
| --- | --- | --- |
| Organization or company | 14 | [33, 38, 39, 41, 45, 61, 67, 70, 86, 99, 101, 104, 106, 107] |
| University or teaching institute | 71 | [9–11, 19, 21, 25–37, 40, 43, 44, 47–53, 55–57, 59–65, 69, 71–77, 79–85, 87–93, 95–98, 100–103, 105, 110–112] |
| Research institution | 5 | [6, 33, 66, 89, 98] |
| Journal | 2 | [16, 46] |
| Insurance company | 1 | [109] |
| Government agency | 4 | [14, 48, 54, 78] |
| Law firm | 3 | [40, 42, 87] |
| Unknown | 5 | [15, 58, 68, 94, 108] |
